# The association of near-infrared spectroscopy-derived tissue oxygenation measurements with sepsis syndromes, organ dysfunction and mortality in emergency department patients with sepsis

**DOI:** 10.1186/cc10463

**Published:** 2011-09-22

**Authors:** Nathan I Shapiro, Ryan Arnold, Robert Sherwin, Jennifer O'Connor, Gabriel Najarro, Sam Singh, David Lundy, Teresa Nelson, Stephen W Trzeciak, Alan E Jones

**Affiliations:** 1 Department of Emergency Medicine and Center for Vascular Biology Research, Beth Israel Deaconess Medical Center and Harvard Medical School, 330 Brookline Avenue, Boston, MA 02215, USA; 2 Department of Emergency Medicine, Cooper University Hospital and the University of Medicine and Dentistry of New Jersey-Robert Wood Johnson Medical School at Camden, One Cooper Plaza, Camden, NJ 08103, USA; 3 Department of Emergency Medicine, Detroit Receiving Hospital and Wayne State University, 4201 St Antoine 6G, UHC, Detroit, MI 48201, USA; 4 Department of Emergency Medicine, University of Mississippi Medical Center, 2500 North State Street, Jackson, MS 39211 USA; 5 Technomics Research, LLC, 1815 Medina Road, Minneapolis, MN 55356, USA; 6 Department of Critical Care, Cooper University Hospital and University of Medicine and Dentistry of New Jersey-Robert Wood Johnson Medical School at Camden, One Cooper Plaza, Camden, NJ 08103, USA

## Abstract

**Introduction:**

Near-infrared spectroscopy (NIRS) noninvasively measures peripheral tissue oxygen saturation (StO_2_). NIRS may be utilized along with a vascular occlusion test, in which limb blood flow is temporarily occluded and released, to quantify a tissue bed's rate of oxygen exchange during ischemia and recovery. The objective of this study was to test the hypothesis that NIRS-derived StO_2 _measures (StO_2 _initial, StO_2 _occlusion and StO_2 _recovery) identify patients who are in shock and at increased risk of organ dysfunction (Sequential Organ Failure Assessment (SOFA) score ≥ 2 at 24 hours) and dying in the hospital.

**Methods:**

This prospective, observational study comprised a convenience sample of three cohorts of adult patients (age > 17 years) at three urban university emergency departments: (1) a septic shock cohort (systolic blood pressure < 90 after fluid challenge; the "SHOCK" cohort, *n *= 58), (2) a sepsis without shock cohort (the "SEPSIS" cohort, *n *= 60) and emergency department patients without infection (*n *= 50). We measured the StO_2 _initial, StO_2 _occlusion and StO_2 _recovery slopes for all patients. Outcomes were sepsis syndrome severity, organ dysfunction (SOFA score at 24 hours) and in-hospital mortality.

**Results:**

Among the 168 patients enrolled, mean initial StO_2 _was lower in the SHOCK cohort than in the SEPSIS cohort (76% vs 81%), with an impaired occlusion slope (-10.2 and 5.2%/minute vs -13.1 and 4.4%/minute) and an impaired recovery slope (2.4 and 1.6%/second vs 3.9 and 1.7%/second) (*P *< 0.001 for all). The recovery slope was well-correlated with SOFA score at 24 hours (-0.35; *P *< 0.001), with a promising area under the curve (AUC) for mortality of 0.81. The occlusion slope correlation with SOFA score at 24 hours was 0.21 (*P *< 0.02), with a fair mortality AUC of 0.70. The initial StO_2 _was significantly but less strongly correlated with SOFA score at 24 hours (-0.18; *P *< 0.04), with a poor mortality AUC of 0.56.

**Conclusions:**

NIRS measurements for the StO_2 _initial, StO_2 _occlusion and StO_2 _recovery slope were abnormal in patients with septic shock compared to sepsis patients. The recovery slope was most strongly associated with organ dysfunction and mortality. Further validation is warranted.

**Trial registration:**

NCT01062685

## Introduction

Severe sepsis currently accounts for > 500,000 emergency department (ED) visits [1] and over 750,000 cases annually in the United States [2]. While the etiologies and presentations of sepsis remain extremely heterogeneous, the disease pathophysiology comprises a dysregulated host response, activation of the inflammatory and coagulation cascades, tissue hypoxia, cellular dysfunction, organ dysfunction and, if unmitigated, death. Though biological questions and controversy remain, there is widespread support for the basic principle that early identification and timely supportive care, coupled with antibiotic therapy and source control, result in improved outcomes. As a result, current international consensus guidelines for the resuscitation of patients with severe sepsis and septic shock recommend aggressive, invasive, protocol-directed care titrating to centrally monitored parameters [3]. Unfortunately, central monitoring is not uniformly available and is often cited as a barrier to guideline compliance [4]. A noninvasive and reproducible measure of tissue hypoxia would be a valuable asset in the resuscitation armamentarium.

**Figure 1:**
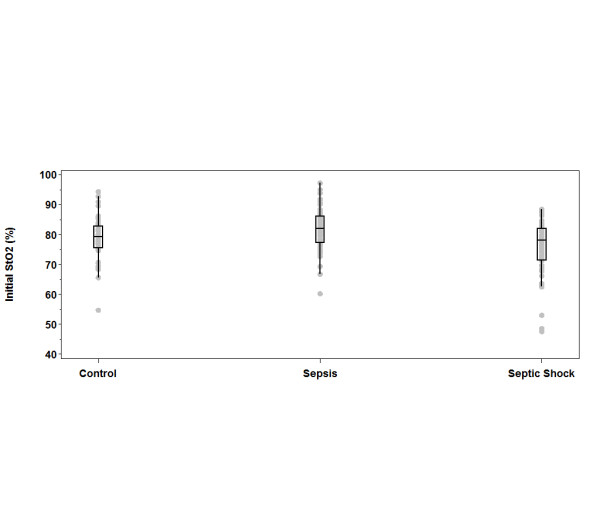
**Tissue oxygen saturation vasoocclusive testing**. The initial slope, occlusion slope and recovery slopes are shown. During the initial phase, the tissue oxygen saturation (StO_2_) level is monitored over time (initial). At occlusion, the tourniquet is programmed to insufflate a cuff to 50 mmHg above the patient's systolic blood pressure. The StO_2 _level is then monitored during the ischemic period to calculate the steady-state ischemic downslope (ischemic slope). This is postulated to represent a combination of oxygen content in the microcirculation and the metabolic demand of the tissues. Next the cuff is released so that blood flow may be reestablished. The tissue is then reperfused, with the rate representing the capacity to autoregulate and reperfuse the tissue exposed to regional ischemia (recovery slope). A patient with intact endothelial cell function, microcirculation and oxygenation capacity will reperfuse quickly, yielding a steep recovery slope, while patients in whom these mechanisms are impaired will have a shallower recovery slope.

One option for noninvasive assessment of tissue hypoxia is near-infrared spectroscopy (NIRS), which first entered the medical field in 1977 as a method for measuring oxygen levels in muscle and other tissues *in vivo *[5]. With NIRS, it is possible to assess the ratio of oxygenated to deoxygenated hemoglobin, resulting in an indirect measure of tissue oxygenation. NIRS has shown promise as a tool to assess tissue oxygenation in a number of settings, including trauma, congestive heart failure and sepsis. Its true diagnostic value and specific interventional role for guiding therapy require further study, however. Additionally, the use of NIRS in conjunction with vasoocclusive testing (VOT) is a tool with the capacity to assess endothelial cell function, microcirculatory capacity and autoregulatory reserve (Figure 1). Further study of the VOT procedure is required to establish its true utility as prognostic indicator and an end point of resuscitation.

To evaluate the utility of the aforementioned NIRS parameters, we conducted an ED-based study of patients presenting across a spectrum of sepsis severities, along with age and sex matched non-infected control patients. There are three main NIRS measurements reported in the literature: (1) continuous tissue oxygen saturation (StO_2_) measurement (StO_2 _initial), (2) StO_2 _occlusion slope (StO_2 _downslope) in response to VOT testing and 3) StO_2 _recovery slope (StO_2 _upslope) in response to VOT. In this study, we assessed the association of each of these parameters with severity of illness, organ dysfunction and death. More specifically, the objective of this study was to test the hypothesis that NIRS-derived StO_2 _measures (StO_2 _initial, StO_2 _occlusion and StO_2 _recovery) are able to identify patients who are in shock and at increased risk of organ dysfunction (Sequential Organ Failure Assessment (SOFA) [6] score ≥ 2 at 24 hours) and dying in the hospital.

## Materials and methods

### Study design and overall approach

We conducted a prospective, multicenter, observational study of a convenience sample of patients who presented to the ED of one of three large, urban, tertiary care facilities. Three specific cohorts of patients (septic shock (the "SHOCK" cohort), sepsis without shock (the "SEPSIS" cohort) and uninfected controls) were assembled, encompassing a spectrum of sepsis severity. We collected pertinent demographic and clinical covariates as well as initial StO_2_% and NIRS-derived measurements in response to VOT testing. Then we analyzed the association and predictive ability of the NIRS measurements with our outcomes of interest. The study was approved by the ethics committees of each of the hospitals.

### Assembly of cohorts

Three distinct cohorts of patients were enrolled. The SHOCK cohort had to meet the American College of Chest Physicians/Society of Critical Care Medicine criteria for septic shock, specifically (1) suspected infection, (2) fulfillment of two or more of the criteria for systemic inflammatory response syndrome (SIRS) (temperature > 100.4°F or < 96.8°F, heart rate > 90 beats/minute, respiratory rate > 20 breaths/minute or partial pressure of carbon dioxide < 32 mmHg, white blood cell count > 12,000/μL or < 4,000/μL or > 10% bands) and (3) hypotension despite adequate fluid resuscitation (systolic blood pressure (SBP) < 90 mmHg after 20 mL/kg crystalloids) [7]. The SEPSIS cohort had to meet the inclusion criteria of suspected infection, two or more SIRS criteria (see above) and no refractory hypotension. The third cohort comprised uninfected ED control patients who met the criteria of no suspected infection, no SIRS criteria met and no evidence of hypoperfusion. The control patients were age-matched (by decade) as well as sex- and race-matched to the SHOCK cohort.

A common set of exclusion criteria were applied to all patient cohorts, which included any of the following: age < 18 years, pregnancy, established "Do Not Resuscitate" orders prior to enrollment, acute traumatic or burn injury (primary diagnosis), acute cerebrovascular event (primary diagnosis), acute coronary syndrome (primary diagnosis), acute pulmonary edema (primary diagnosis), cardiac dysrhythmia (primary diagnosis), acute and active gastrointestinal bleeding (primary diagnosis), acute drug overdose (primary diagnosis), requirement for immediate surgery and inability to obtain written informed consent. Clinical management at each institution is in agreement with the Surviving Sepsis Campaign guidelines.

### Demographic and clinical covariates

We collected demographic variables (age, sex and race), comorbidities (cerebrovascular disease, chemotherapy, congestive heart failure, chronic obstructive pulmonary disease, dementia, diabetes, HIV or AIDS, Hodgkin's disease, intravenous drug use, leukemia, liver disease, myocardial infarction, non-Hodgkin's lymphoma, peripheral vascular disease, renal disease, splenectomy, steroid use, ulcer disease, transplant, presence of any malignancy and residence in a nursing home), vital signs data (temperature, blood pressure, heart rate, respiratory rate and oxygen saturation) and pertinent laboratory data (serum lactate, complete blood count, chemistry panels, markers of coagulation and liver function tests).

### NIRS assessment

The InSpectra StO_2 _Tissue Oxygenation Monitor (model 650; Hutchinson Technology, Hutchinson, MN, USA) with probes spaced at 15 mm was utilized to obtain StO_2 _measurements. The measurements were taken at the thenar eminence during the resuscitation phase. Following a minimum initial five-minute stabilization period, we assessed the initial StO_2 _measurement and then performed a VOT procedure using an automated tourniquet (Delfi Tourniquet System; Delfi Medical Innovations, Inc, Vancouver, BC, Canada), which was insufflated to 50 mmHg over the patient's SBP for a period of three minutes. After three minutes, the cuff was quickly removed. The subsequent StO_2 _tracing was analyzed offline to record the following NIRS-derived metrics (see Figure 1) (1) StO_2 _initial, the baseline StO_2 _recorded after a five-minute stabilization period; (2) StO_2 _occlusion, the steady-state rate of occlusion (StO_2_%/second), represented by the descending slope during the ischemic period; and (3) StO_2 _recovery, the steady-state recovery slope during the reoxygenation phase after the tourniquet was released. The StO_2 _measurements were imported into a Microsoft Excel software file (Microsoft Corporation, Redmond, WA, USA), and the slopes were derived by (1) drawing a best-fit line for the steady-state slope for the respective metric and (2) calculating the slope.

### Outcomes

We examined the association of StO_2 _parameters in relation to three patient-oriented outcomes: (1) presence of shock, as defined above, assessed at the time of enrollment; (2) in-hospital mortality, defined as vital signs status at hospital discharge; and (3) organ dysfunction at 24 hours assessed on the basis of the SOFA scores calculated at the time of enrollment and 24 hours later [6]. Consistent with prior publications, we defined organ dysfunction as a SOFA score ≥ 2, which was our primary outcome of interest. The use of a threshold SOFA score ≥ 2 for an ill patient has previously been established [8-10]. All patients in the control group with missing SOFA scores at 24 hours (discharge was the primary reason for missing data) were assumed to have a SOFA score < 2. For patients enrolled with a history of chronic renal insufficiency or end-stage renal disease, the renal SOFA score was not included in the total SOFA score.

### Data analysis

Descriptive statistics (means, standard deviations, medians or proportions with percentiles) were reported for demographics, clinical characteristics, vital signs and laboratory values stratified by the three cohorts. We compared mean (or median) values for the StO_2 _parameters of interest (initial, ischemic slope and reperfusion slope) of the three different groups using the Wilcoxon two-sample test. We used a Bonferroni correction to address multiple testing; thus, for this analysis, Cronbach's α < 0.017 was considered significant. Next, we compared the StO_2 _parameters of interest for the outcomes of in-hospital mortality and SOFA scores ≥ 2 at 24 hours. To assess the diagnostic accuracy for the StO_2 _parameters as a predictor of outcomes, we constructed receiver operating characteristic curves (ROCs) and calculated the area under the curve (AUC) along with the 95% confidence intervals (95% CIs). We used multivariate logistic regression models to obtain adjusted estimates for age, serum lactate and SBP and to identify the StO_2 _parameters and adjustor variables with the strongest independent associations with outcomes by using a stepwise backward elimination technique with forward examination of parameters eliminated after final model selection. Throughout the analysis we used serum lactate level as a comparison predictor.

### Sample size calculation

Our study was powered on the ability of our anticipated best StO_2 _readout (StO_2 _recovery slope) to discriminate the SEPSIS cohort from the SHOCK cohort. Based on previous studies, assuming mean changes in slope of 2.3 ± 1.3 for the SHOCK group and 3.2 ± 1.4 for the SEPSIS group, with a power of 90% and Cronbach's α set at 0.05, we calculated that approximately 60 patients per group were needed [11]. We also enrolled 50 uninfected controls as comparators for comparisons between controls and the sepsis groups.

## Results

### Patient characteristics

We enrolled 170 patients in the study. However, two patients in the SHOCK group were withdrawn from the study (one voluntary withdrawal and one with incomplete StO_2 _data), leaving a total of 168 patients in the study group. Of these, 58 had septic shock upon enrollment, 60 had sepsis without shock and 50 were uninfected control patients. The mortality rates were 38% for the SHOCK cohort, 5% for the SEPSIS cohort and 0% for the control group. The overall mean age for the population was 63 years, of whom 60% were males (Table 1). The SHOCK patients were older than the SEPSIS patients but similar in age to the control patients, since we matched these groups for age and sex. The distribution of comorbidities was similar in the three groups, but, as expected, the clinical characteristics (for example, blood pressure) and laboratory values (for example, serum lactate) commonly associated with increased severity of illness were worse in the SHOCK group.

**Table 1:** Selected demographic and medical history variables^a^

Patient demographic variables	Group (*N *= 168)		
	SHOCK (*n *= 58)	SEPSIS (*n *= 60)	Control (*n *= 50)
Age, years	68 ± 16 (72)	55 ± 17 (52)	68 ± 16 (72)
Weight, lb	180 ± 58 (175)	194 ± 59 (184)	165 ± 41 (165)
Male sex, *n *(%)	38 (66%)	38 (63%)	24 (48%)
History of coronary artery disease, *n *(%)	17 (29%)	13 (22%)	12 (24%)
Myocardial Infarction, *n *(%)	8 (14%)	5 (8%)	6 (12%)
Hypertension, *n *(%)	34 (59%)	33 (55%)	28 (56%)
Chronic obstructive pulmonary disease, *n *(%)	12 (21%)	10 (17%)	6 (12%)
Diabetes (any), *n *(%)	19 (33%)	17 (28%)	14 (28%)
Peripheral vascular disease, *n *(%)	3 (5%)	1 (2%)	4 (8%)
Renal disease, *n *(%)	15 (26%)	8 (13%)	10 (20%)
Serum lactate, mmol/L	3.5 ± 2.5 (3.0), 0.6 to 12.5	1.7 ± 1.1 (1.6), 0.4 to 5.2	1.4 ± 0.7 (1.1), 0.6 to 3.2
Systolic blood pressure, mmHg	100 ± 26 (99)	123 ± 26 (123)	138 ± 28 (138)
Mean arterial pressure, mmHg	75 ± 19 (69), 32 to 127	89 ± 16 (88), 65 to 129	96 ± 16 (96), 62 to 136
Respiratory rate, breaths/minute	26 ± 12 (24), 7 to 95	22 ± 6 (20), 14 to 42	20 ± 12 (18), 13 to 98
SaO_2_, %	97 ± 2 (98)	97 ± 3 (97)	96 ± 6 (97)
Temperature, °F	99.8 ± 3.1 (100.2)	100.0 ± 2.0 (100.3)	97.4 ± 0.8 (97.4)
White blood cell count, ×10^3^/μL	14.6 ± 9.9 (12.2)	13.5 ± 9.6 (12.7)	7.1 ± 2.3 (6.6)

^a^SHOCK: the septic shock cohort (systolic blood pressure < 90 mmHg after fluid challenge); SEPSIS: the sepsis without shock cohort; Control: emergency department patients without infection; SaO_2_: oxygen saturation of arterial blood. Data are means ± SD (medians) unless otherwise indicated.

### Differentiation of SHOCK, SEPSIS and control cohorts

**Figure 2:**
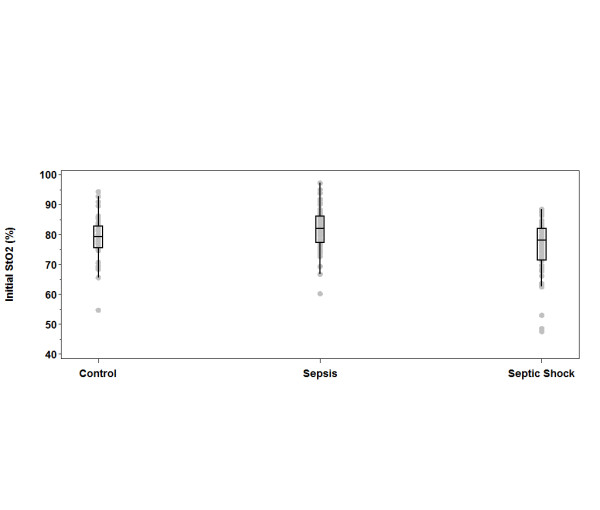
**Differences in initial, ischemic and recovery slopes stratified by sepsis severity**. The boxplots are for initial tissue oxygen saturation (StO_2_). The top and bottom lines of the box are the 25th and 75th percentiles, respectively. The middle line is the median. The whiskers extend to the last data point within 1.5 quartile ranges of the box. The gray dots are the individual observed data points.

The mean values for the three main StO_2 _parameters of interest, StO_2 _initial, ischemic slope and reperfusion slope, were calculated and compared for the different levels of sepsis syndrome and for controls upon enrollment (Table 2 and Figures 2 through 4). The mean values for all three parameters in the SHOCK patients were significantly different from those in the SEPSIS patients. However, the comparison between the SHOCK patients and controls (who were age- and sex-matched) revealed that only the upslope mean was significantly different in the SHOCK vs control cohorts. Conversely, the initial and ischemic slope means, but not the recovery slope mean, were significantly different between the SEPSIS and control groups.

**Table 2:** Vasoocclusive testing parameters at initial presentation^a^

StO_2 _parameters	Groups (*N *= 168)			*P *values		
	SHOCK (*n *= 58)	SEPSIS (*n *= 60)	Control (*n *= 50)	SHOCK vs SEPSIS	SHOCK vs control	SEPSIS vs control
Initial, %	76 ± 9 (78)	82 ± 7 (82)	79 ± 7 (79)	< 0.001*	0.23	< 0.02
Occlusion, %/second	-10.2 ± 5.2 (-9.6)	-13.1 ± 4.4 (-13.1)	-11.3 ± 4.5 (-10.8)	< 0.002*	0.21	< 0.03
Recovery, %/second	2.4 ± 1.6 (2.0)	3.9 ± 1.7 (4.1)	3.8 ± 1.7 (3.8)	< 0.001*	< 0.001*	0.52

^a^SHOCK: the septic shock cohort (systolic blood pressure < 90 mmHg after fluid challenge); SEPSIS: the sepsis without shock cohort; Control: emergency department patients without infection; StO_2_: tissue oxygen saturation; *Wilcoxon two-sample test; statistically significant using *P *= 0.017 with the Bonferroni correction. Data are means ± SD (medians).

**Figure 3:**
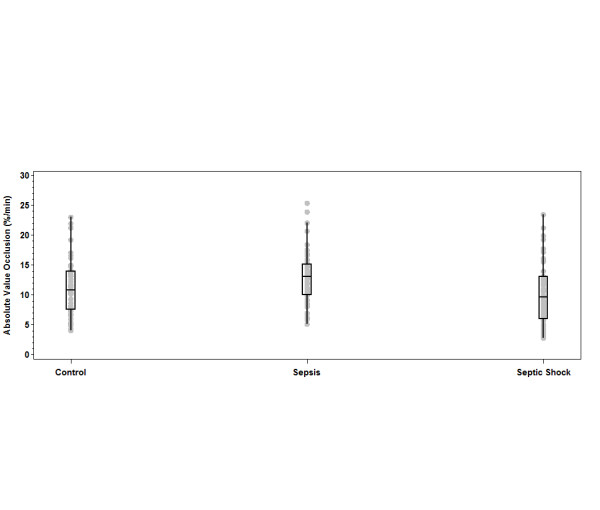
**Differences in initial, ischemic and recovery slopes stratified by sepsis severity**. Ischemic slope. The top and bottom lines of the box are the 25th and 75th percentiles. The middle line is the median. The whiskers extend to the last data point within 1.5 quartile ranges of the box. The gray dots are the individual observed data points.

**Figure 4:**
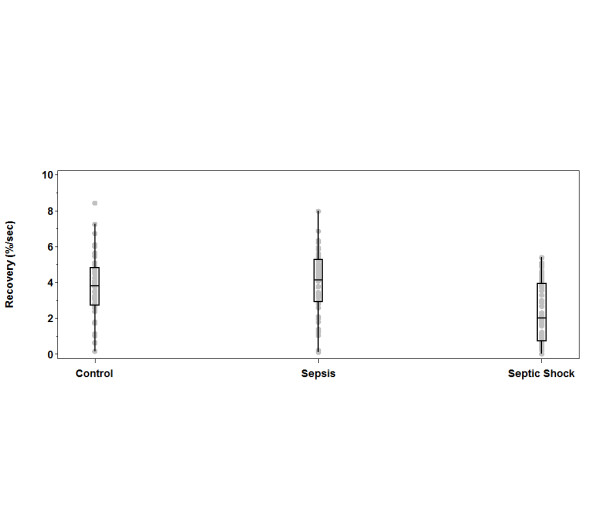
**Differences in initial, ischemic and recovery slopes stratified by sepsis severity**. Recovery slope. The top and bottom lines of the box are the 25th and 75th percentiles. The middle line is the median. The whiskers extend to the last data point within 1.5 quartile ranges of the box. The gray dots are the individual observed data points.

### Mortality prediction

**Figure 5:**
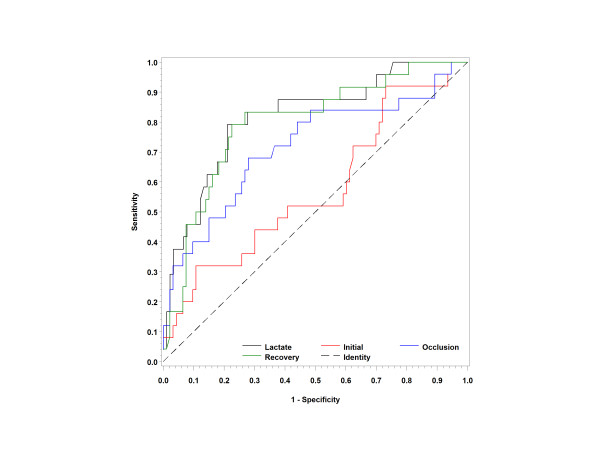
**Receiver operating characteristic curves for mortality**. The receiver operating characteristic (ROC) curves for the initial StO_2_, ischemic slope, recovery slope and serum lactate measurements as predictors of in-hospital mortality are shown.

For the mortality outcomes, we assessed the StO_2 _parameters obtained in the ED, as well as serum lactate, SBP and age. The recovery slopes for patients who died were significantly lower (mean ± SD: 1.7 ± 1.5 vs StO_2_%: 3.7%/second; *P *< 0.0001), with impaired oxygen recovery observed among the nonsurvivors (Table 3). Similarly, the ischemic slope was less steep, showing decreased oxygen consumption during the vasoocclusion phase of the VOT (mean ± SD: -8.8 ± 5.1 vs StO_2_%: -12.0 ± 4.7%/second; *P *< 0.002). Both of these metrics are postulated to represent impaired microcirculation and a reduced capacity to exchange and deliver oxygen. Initial StO_2_% did not differ significantly between the survivors and nonsurvivors, nor did the mortality rate differ when StO_2 _was stratified as < 80% or ≥ 80% (15% vs 15%; *P *= 1.0). The AUC as a predictor of mortality was 0.81 (95% confidence interval: 0.71 to 0.91) for the recovery slope, 0.70 (0.57 to 0.83) for the ischemic slope and 0.56 (0.43 to 0.69) for the initial slope. Serum lactate levels were also increased in the nonsurvivors compared to the survivors (4.7 ± 2.7 vs 1.9 ± 1.4 mmol/L; *P *< 0.001), with an AUC of 0.85 for serum lactate. The ROCs are shown in Figure 5. The multivariable logistic regression model used to determine independent predictors of mortality included the age, serum lactate, SBP and StO_2 _parameters. Using both forward and backward selection techniques, the lactate and recovery slopes were retained in the model as the strongest predictors of in-hospital mortality, regardless of cohort. These two variables yielded an AUC for model discrimination of 0.88.

**Table 3:** Serum lactate, systolic blood pressure and InSpectra parameters stratified by in-hospital survival in all groups^a^

Parameter	Died (*n *= 25)	Survived (*n *= 143)	*P *value*	AUC (95% CI)
Serum lactate, mmol/L	4.7 ± 2.7 (4.2) (*n *= 24)	1.9 ± 1.4 (1.5) (*n *= 128)	< 0.001	0.85 (0.76 to 0.93)
Systolic blood pressure, mmHg	105 ± 30 (102)	122 ± 30 (123)	0.004	0.68 (0.56 to 0.80)
Mean age, years	69 ± 14 (72)	62 ± 18 (67)	0.100	0.60 (0.49 to 0.72)
StO_2_				
Initial (%)	76 ± 11 (79)	79 ± 7 (80)	0.341	0.56 (0.43 to 0.69)
Occlusion (%/minute)	-8.8 ± 5.1 (-8.2)	-12.0 ± 4.7 (-11.9)	0.002	0.70 (0.57 to 0.83)
Recovery (%/second)	1.7 ± 1.5 (1.3)	3.7 ± 1.7 (3.8)	< 0.001	0.81 (0.71 to 0.91)

^a^AUC: area under the curve; 95% CI: 95% confidence interval; StO_2_: tissue oxygen saturation; *Wilcoxon two-sample test or Fisher's exact test, as appropriate. Data are means ± SD (medians).

### Organ dysfunction at 24 hours

**Figure 6:**
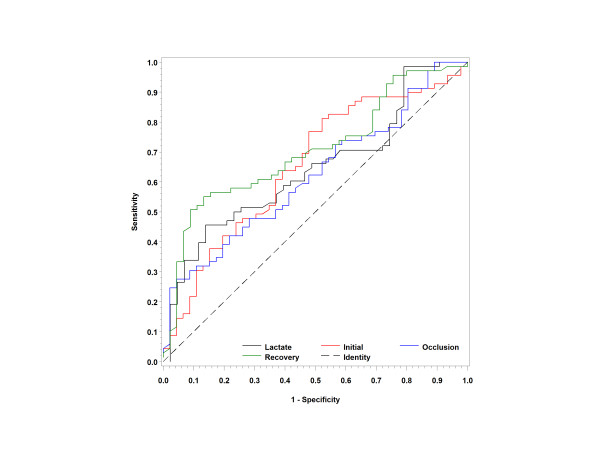
**Receiver operating characteristic curves for Sequential Organ Failure Assessment scores ≥ 2**. The receiver operating characteristic (ROC) curves for the initial tissue oxygen saturation, ischemic slope, recovery slope and serum lactate measurements as a predictor of Sequential Organ Failure Assessment scores > 2 at 24 hours are shown. The dashed line represents an AUC of 0.5 consistent with purely due to chance alone.

Next, we assessed the StO_2 _parameters for their ability to predict organ dysfunction, defined *a priori *as SOFA score ≥ 2 at 24 hours. The initial and occlusion StO_2 _metrics, as well as serum lactate, SBP and age, were significantly abnormal in patients with SOFA scores ≥ 2 at 24 hours compared to those with SOFA scores < 2 (Tables 4 and 5). The StO_2 _occlusion slope did not show a statistically significant difference between the groups. The AUC for the groups were initial slope, 0.61 (0.52 to 0.70); ischemic slope, 0.57 (0.48 to 0.66); recovery slope 0.68 (0.59 to 0.76); and lactate slope 0.69 (0.61 to 0.78). The ROCs are shown in Figure 6. We also examined the correlation between the NIRS parameters at initial presentation and the total SOFA score and found a correlation between StO_2 _initial slope (Spearman's ρ correlation coefficient = -0.18; *P *< 0.04), occlusion slope (Spearman's ρ correlation coefficient = 0.21; *P *< 0.02) and recovery slope (Spearman's ρ correlation coefficient = -0.35; *P *< 0.001).

**Table 4:** Serum lactate and systolic blood pressure- and near-infrared spectroscopy-derived parameters stratified by Sequential Organ Failure Assessment score at 24 hours for all groups^a^

Parameter	SOFA score ≥ 2 at 24 hours^#^			AUC (95% CI)
	Yes (*n *= 65)	No (*n *= 100)	*P *value*	
Serum lactate, mmol/L	3.1 ± 2.2 (2.1)	1.7 ± 1.5 (1.5)	< 0.001	0.69 (0.61 to 0.78)
Systolic blood pressure	107 ± 27 (104)	128 ± 30 (126)	< 0.001	0.71 (0.63 to 0.79)
Mean age, years	67 ± 17 (71)	61 ± 18 (64)	0.020	0.61 (0.52 to 0.70)
StO_2_				
Initial (%)	77 ± 9 (79)	80 ± 7 (81)	< 0.02	0.61 (0.52 to 0.70)
Occlusion (%/minute)	-10.7 ± 4.9 (-10.6)	-12.1 ± 4.8 (-11.6)	0.14	0.57 (0.48 to 0.66)
Recovery (%/second)	2.7 ± 1.9 (2.0)	3.8 ± 1.7 (3.9)	< 0.001	0.68 (0.59 to 0.76)

^a^SOFA: Sequential Organ Failure Assessment; AUC: area under the curve; 95% CI: 95% confidence interval; StO_2_: tissue oxygen saturation. ^#^For patients in the control group in which the SOFA score at 24 hours was not measured, a SOFA score < 2 was assumed. For patients with a history of chronic renal insufficiency or end-stage renal disease, the total SOFA score used did not include the renal portion of the score. *Wilcoxon two-sample test or Fisher's exact test, as appropriate. Data are means ± SD (medians).

**Table 5:** Results of multivariate logistic regression modeling for the outcomes of Sequential Organ Failure Assessment score ≥ 2 at 24 hours and in-hospital mortality in all groups^a^

Parameter included	Parameter retained (Yes/No (*P *value))^b^	
	Outcomes	
	SOFA ≥ 2 at 24 hours^c^	In-hospital mortality
Age, years	No (0.086)	No (0.089)
Serum lactate	Yes (< 0.001)	Yes (< 0.001)
Systolic blood pressure	Yes (0.014)	No (0.571)
StO_2_		
Initial	Yes (0.046)	No (0.128)
Occlusion	No (0.199)	No (0.669)
Recovery	Yes (0.037)	Yes (0.003)
Multivariate model AUC (95% CI)	0.79 (0.72 to 0.87)	0.88 (0.80 to 0.95)
Log (*p*/(1 - *p*))	5.6651 + 0.5135*Lactate - 0.0173*SBP - 0.0529*Initial - 0.2465*Recovery	-1.7582 + 0.5084*Lactate - 0.5271*Recovery
Odds ratios (95% CI)		
Serum lactate (1 mEq/L increase)	1.7 (1.2 to 2.2)	1.7 (1.3 to 2.2)
SBP (10 mmHg decrease)	1.2 (1.0 to 1.4)	NA
Initial (10% decrease)	1.7 (1.0 to 2.9)	NA
Upslope (1%/second decrease)	1.3 (1.0 to 1.6)	1.7 (1.2 to 2.4)

^a^SOFA: Sequential Organ Failure Assessment; StO_2_: tissue oxygen saturation; AUC: area under the curve; 95% CI: 95% confidence interval; SBP: systolic blood pressure; NA: not applicable. ^b^Stepwise backward elimination technique used. The *P *value in parentheses after "Yes" is the *P *value in the resultant model. The *P *value after "No" is the *P *value when the eliminated parameter was included back into the final model for reevaluation after elimination. ^c^For patients in the control group in which the SOFA score at 24 hours was not measured, a SOFA score < 2 was assumed. For patients with a history of chronic renal insufficiency or end-stage renal disease, the total SOFA score used did not include the renal portion of the score.

### Sensitivity analysis

We chose to use the entire study population and included the uninfected control population in our analyses to assess mortality outcomes, as well as organ dysfunction at 24 hours, to take advantage of our full data set. However, one could argue against this approach and make the case for including only patients who fulfilled the minimum inclusion criteria for the definition of sepsis. Thus, we performed a subsequent sensitivity analysis in which we limited the analysis to the 118 patients enrolled from the SEPSIS and SHOCK groups. The results of this analysis were very similar to those of our primary analyses (Additional file 1 online data supplement).

## Discussion

In this multicenter study of ED patients who presented across the spectrum of sepsis illness severity, we have demonstrated that there is a potential role for noninvasive NIRS technology in patient assessment and risk stratification. Moreover, we found that the use of NIRS in conjunction with VOT testing, specifically as assessed on the basis of the recovery slope, may hold the most promise for use in the ED. The accuracy of recovery slopes measured in the ED was similar to serum lactate measurements with regard to the prediction of mortality as well as organ dysfunction at 24 hours. These findings remained robust in multivariate models. Additionally, in the evolving era of goal-directed resuscitation protocols, further investigation is needed to determine whether NIRS, in combination with VOT testing, has a role in guiding therapeutic efforts and has promise as a noninvasive assessment of tissue oxygenation.

Our findings that VOT testing helped to optimize NIRS diagnostic utility are consistent with the rationale that measuring the body's capacity to reoxygenate tissue in response to the physiological perturbation of induced ischemia is a valid method of assessing an individual's physiological function and reserve capacity. VOT is a procedure whereby, for a limited time period (for example, three minutes), blood flow to the muscle is interrupted by using a tourniquet, allowing tissue desaturation to occur. The ischemic tissue then induces vasodilation of surrounding arterioles, metarterioles and precapillary sphincters to decrease local vascular resistance and regain blood flow. Next, as the tourniquet is released and blood flow is restored, there is a reactive hyperemic response which represents the tissue's ability to autoregulate blood flow and oxygenation [12]. The speed at which tissues are reoxygenated is proposed to represent the reserve capacity and functionality of the endothelium, mitochondria and microcirculation. Thus, in the simplest terms, flow will quickly be restored in a patient with intact autoregulatory capacity, resulting in a steep recovery slope. A patient with dysfunction in any of these components will manifest impaired reoxygenation and a shallower recovery slope.

In fact, researchers in prior studies have reported similar results. For example, in a 90-patient ICU-based study (plus 18 healthy volunteers), Creteur *et al. *[11] showed a significant association between a reduced reperfusion slope after VOT testing and both shock and mortality. The recovery slope outperformed the other NIRS-derived variables. Payen *et al. *[13] also found a depressed reperfusion slope in septic shock patients, as did Skarda *et al. *[14]. Other researchers who have used VOT testing have found increased StO_2 _recovery times in patients with hemorrhagic shock [15], septic shock [14] and peripheral vascular disease [16], including patients in whom initial (preocclusion) StO_2 _readings were high, > 75%. Accordingly, we submit that a primary message of our present study is that VOT testing in conjunction with NIRS might hold the most diagnostic potential.

While our results offer enthusiasm for the use of NIRS-derived StO_2 _parameters, especially the reperfusion slope, to predict adverse outcomes and potentially guide resuscitation, some of our findings are not as promising and warrant further investigation. For example, the StO_2 _initial slope (StO_2 _without VOT) and the occlusion slope were unable to discriminate patients in septic shock from age-, sex- and race-matched controls. StO_2 _initially had a poor AUC (0.56) for mortality. The reperfusion slope was not significantly different between patients with sepsis and controls; however, that result was not entirely unexpected, as the conditions of many of the patients meeting the sepsis definition were of low acuity. While the performance of StO_2 _parameters in predicting organ dysfunction were similar to the commonly used marker of serum lactate, the AUCs for these parameters (0.58 to 0.67) showed only fair discrimination.

There are a number of additional limitations of this study, which was designed to be only an initial look at NIRS testing in the ED. We used a convenience sample of patients and recruited a similar number of patients in the SHOCK and SEPSIS groups, as well as an age-, sex- and race-matched control group, which by definition was enrolled in a nonconsecutive manner, thus exposing our study to selection bias. Since we enrolled a skewed population, we did not attempt to identify clinically useful cutoff values that could be validated in future studies. We did not assess the reproducibility of our NIRS measurements, which may threaten the reliability and reproducibility of our overall results. We also measured the slopes manually, which may affect the reproducibility of results. Our patient population included a limited number of deaths, leaving our estimates of this outcome with large 95% CIs. Our outcome measures of sepsis syndrome at the time of enrollment and SOFA scores ≥ 2 have been well-reported, but one may challenge their clinical relevance. In this study, we did not follow changes in StO_2 _measurements over time. There are a number of other StO_2 _measurements that may be derived as part of the VOT procedure that we did not assess.

## Conclusions

We conclude that NIRS-derived measurements, including those that are part of a VOT protocol, hold promise for risk stratification and patient assessment in the ED. Further studies are warranted to assess the reproducibility of our findings and to determine the value of NIRS-derived parameters as end points of a noninvasive resuscitation protocol.

## Key messages

• NIRS-derived StO_2 _measurements hold promise for a role in risk stratification in ED patients with sepsis.

• Critically ill patients with sepsis have a reduced rate of oxygen recovery as measured using NIRS in response to VOT.

• The StO_2 _oxygen recovery slope was the best-performing NIRS parameter, having the highest association with shock, organ dysfunction and death.

## Abbreviations

AUC: area under the curve; ED: emergency department; NIRS: near infrared spectroscopy; ROC: receiver operating characteristic curve; SOFA: Sequential Organ Failure Assessment; StO_2_: tissue oxygen saturation.

## Competing interests

This study was funded by an investigator-initiated grant from Hutchinson Technology (Hutchinson, MN, USA).

## Authors' contributions

NIS, RA, SWT and AEJ participated in the initial study conception and design. All authors (except TN) participated in the implementation and conduct of the study. TN performed statistical analysis. All authors participated in manuscript preparation. All authors read and approved the final manuscript for publication.

## Supplementary Material

Additional file 1 **Online data supplement: A sensitivity analysis limiting the analysis to the 118 patients from the SEPSIS and SHOCK groups who were enrolled in the study**. Click here for file

## References

[B1] Wang HE Shapiro NI Angus DC Yealy DM National estimates of severe sepsis in United States emergency departments Crit Care Med 2007 35 1928 1936 10.1097/01.CCM.0000277043.85378.C1 17581480

[B2] Angus DC Linde-Zwirble WT Lidicker J Clermont G Carcillo J Pinsky MR Epidemiology of severe sepsis in the United States: analysis of incidence, outcome, and associated costs of care Crit Care Med 2001 29 1303 1310 10.1097/00003246-200107000-00002 11445675

[B3] Dellinger RP Levy MM Carlet JM Bion J Parker MM Jaeschke R Reinhart K Angus DC Brun-Buisson C Beale R Calandra T Dhainaut JF Gerlach H Harvey M Marini JJ Marshall J Ranieri M Ramsay G Sevransky J Thompson BT Townsend S Vender JS Zimmerman JL Vincent JL International Surviving Sepsis Campaign Guidelines Committee American Association of Critical-Care Nurses American College of Chest Physicians American College of Emergency Physicians Canadian Critical Care Society European Society of Clinical Microbiology and Infectious Diseases European Society of Intensive Care Medicine European Respiratory Society International Sepsis Forum Japanese Association for Acute Medicine Japanese Society of Intensive Care Medicine Society of Critical Care Medicine Society of Hospital Medicine Surgical Infection Society World Federation of Societies of Intensive and Critical Care Medicine Surviving Sepsis Campaign: international guidelines for management of severe sepsis and septic shock: 2008 Crit Care Med 2008 36 296 327 10.1097/01.CCM.0000298158.12101.41 18158437

[B4] Jones AE Kline JA Use of goal-directed therapy for severe sepsis and septic shock in academic emergency departments Crit Care Med 2005 33 1888 1890 10.1097/01.CCM.0000166872.78449.B1 16096485

[B5] Hamaoka T McCully KK Quaresima V Yamamoto K Chance B Near-infrared spectroscopy/imaging for monitoring muscle oxygenation and oxidative metabolism in healthy and diseased humans J Biomed Opt 2007 12 062105 10.1117/1.2805437 18163808

[B6] Vincent JL Moreno R Takala J Willatts S De Mendonça A Bruining H Reinhart CK Suter PM Thijs LG The SOFA (Sepsis-related Organ Failure Assessment) score to describe organ dysfunction/failure. On behalf of the Working Group on Sepsis-Related Problems of the European Society of Intensive Care Medicine Intensive Care Med 1996 22 707 710 10.1007/BF01709751 8844239

[B7] Bone RC Sibbald WJ Sprung CL The ACCP-SCCM consensus conference on sepsis and organ failure Chest 1992 101 1481 1483 10.1378/chest.101.6.1481 1600757

[B8] Moreno R Vincent JL Matos R De Mendonça A Cantraine F Thijs L Takala J Sprung C Antonelli M Bruining H Willatts S The use of maximum SOFA score to quantify organ dysfunction/failure in intensive care: results of a prospective, multicentre study. Working Group on Sepsis related Problems of the ESICM Intensive Care Med 1999 25 686 696 10.1007/s001340050931 10470572

[B9] Janssens U Graf C Graf J Radke PW Königs B Koch KC Lepper W vom Dahl J Hanrath P Evaluation of the SOFA score: a single-center experience of a medical intensive care unit in 303 consecutive patients with predominantly cardiovascular disorders. Sequential Organ Failure Assessment Intensive Care Med 2000 26 1037 1045 10.1007/s001340051316 11030159

[B10] Balci C Sungurtekin H Gürses E Sungurtekin U APACHE II, APACHE III, SOFA scoring systems, platelet counts and mortality in septic and nonseptic patients in Turkish Ulus Travma Acil Cerrahi Derg 2005 11 29 34 15688265

[B11] Creteur J Carollo T Soldati G Buchele G De Backer D Vincent JL The prognostic value of muscle StO_2 _in septic patients Intensive Care Med 2007 33 1549 1556 10.1007/s00134-007-0739-3 17572876

[B12] Doerschug KC Delsing AS Schmidt GA Haynes WG Impairments in microvascular reactivity are related to organ failure in human sepsis Am J Physiol Heart Circ Physiol 2007 293 H1065 H1071 10.1152/ajpheart.01237.2006 17483235

[B13] Payen D Luengo C Heyer L Resche-Rigon M Kerever S Damoisel C Losser MR Is thenar tissue hemoglobin oxygen saturation in septic shock related to macrohemodynamic variables and outcome? Crit Care 2009 13 Suppl 5 S6 10.1186/cc8004 19951390 PMC2786108

[B14] Skarda DE Mulier KE Myers DE Taylor JH Beilman GJ Dynamic near-infrared spectroscopy measurements in patients with severe sepsis Shock 2007 27 348 353 10.1097/01.shk.0000239779.25775.e4 17414414

[B15] Gómez H Torres A Polanco P Kim HK Zenker S Puyana JC Pinsky MR Use of non-invasive NIRS during a vascular occlusion test to assess dynamic tissue O_2 _saturation response Intensive Care Med 2008 34 1600 1607 10.1007/s00134-008-1145-1 18523754

[B16] Comerota AJ Throm RC Kelly P Jaff M Tissue (muscle) oxygen saturation (StO_2_): a new measure of symptomatic lower-extremity arterial disease J Vasc Surg 2003 38 724 729 10.1016/S0741-5214(03)01032-2 14560221

