# Supplementary material for: The association of near-infrared spectroscopy-derived tissue oxygenation measurements with sepsis syndromes, organ dysfunction and mortality in emergency department patients with sepsis
Source: Crit Care. 2011 Sep 22;15(5):R223. doi: 10.1186/cc10463 (PMC3334769; doi:10.1186/cc10463)
Supplement: Additional file 1 — Online data supplement: A sensitivity analysis limiting the analysis to the 118 patients from the SEPSIS and SHOCK groups who were enrolled in the study. [file cc10463-S1.DOC]

Online Supplement

Sensitivity Analyses not including control group

**Table 3A Lactate, SBP and InSpectra Parameters by In-Hospital Survival in Septic Shock and Sepsis Groups**

| **Parameter** | **Died**  **N=25** | **Survived**  **N=93** | **p-value*** | **AUC**  **(95% CI)^** |
| --- | --- | --- | --- | --- |
| Lactate | 4.7 ± 2.7 (4.2)  N=24 | 2.1 ± 1.6 (1.6)  N=90 | <0.001 | 0.82  (0.72, 0.92) |
| Systolic Blood Pressure (mmHg) | 105 ± 30 (102) | 114 ± 28 (116)  N=92 | 0.096 | 0.61  (0.48, 0.74) |
| Age (years) | 69 ± 14 (72) | 59 ± 18 (61) | 0.018 | 0.66  (0.54, 0.77) |
| **StO2** |  |  |  |  |
| Initial (%) | 76 ± 11 (79) | 80 ± 7 (80) | 0.248 | 0.58  (0.44, 0.71) |
| Occlusion (%/minute) | -8.8 ± 5.1 (-8.2) | -12.5 ± 4.7 (-12.6) | <0.001 | 0.72  (0.59, 0.84) |
| Recovery (%/second) | 1.7 ± 1.5 (1.3) | 3.6 ± 1.7 (3.8) | <0.001 | 0.80  (0.70, 0.90) |

*Kruskal-Wallis Test

**Table 4A Lactate, SBP and InSpectra Parameters by SOFA Score at 24 Hours in Septic Shock and Sepsis Groups**

| **Parameter** | **SOFA ≥ 2 at 24 Hours^#^** | | | **AUC**  **(95% CI)** |
| --- | --- | --- | --- | --- |
|  | **Yes**  **N=65** | **No**  **N=50** | **p-value** |  |
| Lactate | 3.1 ± 2.2 (2.1) | 2.0 ± 1.9 (1.6)  N=46 | 0.012 | 0.64  (0.54, 0.74) |
| Systolic Blood Pressure | 107 ± 27 (104) | 117 ± 29 (118) | 0.048 | 0.61  (0.50, 0.71) |
| Age (years) | 67 ± 16 (71) | 54 ± 17 (51) | <0.001 | 0.73  (0.64, 0.82) |
| **StO2** |  |  |  |  |
| Initial (%) | 77 ± 9 (79) | 82 ± 7 (82) | <0.001 | 0.68  (0.58, 0.78) |
| Occlusion (%/minute) | -10.7 ± 4.9  (-10.6) | -12.8 ± 5.0  (-12.7) | 0.053 | 0.61  (0.50, 0.71) |
| Recovery (%/second) | 2.7 ± 1.9 (2.0) | 3.8 ± 1.6 (4.0) | <0.001 | 0.68  (0.58, 0.78) |

^#^For patients with a history of chronic renal insufficiency or end stage renal disease, the total SOFA score used did not include the renal portion of the score.

*Wilcoxon Two-Sample Test

^+^Fisher’s Exact Test

Table 5A Results of Multivariate Logistic Regression Modeling for the Outcomes of SOFA ≥ 2 at 24 Hours and In-Hospital Mortality in Septic Shock and Sepsis Groups

| **Parameter Included** | **Parameter Retained (Yes / No (p-value))^1^** | |
| --- | --- | --- |
|  | **Outcome** | |
|  | **SOFA ≥ 2 at 24 Hours^#^** | **In-Hospital Mortality** |
| Age (years) | Yes (<0.001) | Yes (0.052) |
| Lactate | No (0.069) | Yes (0.003) |
| Systolic Blood Pressure | No (0.275) | No (0.957) |
| StO2 |  |  |
| Initial | Yes (0.022) | No (0.257) |
| Occlusion | Yes (0.018) | No (0.790) |
| Recovery | No (0.242) | Yes (0.018) |
| **Multivariate Model AUC (95% CI)** | 0.78 (0.70, 0.86) | 0.88 (0.81, 0.95) |
| **Log (p/(1-p)** | 4.9156 + 0.0474*Age - 0.0788*Initial StO2 + 0.1049*Occlusion | -4.1067 + 0.0386*Age + 0.4584*Lactate – 0.4501*Recovery |
| Age (10 years increase) | 1.5 (1.1, 2.0) | 1.5 (1.0, 2.2) |
| Lactate (1 mEq/L increase) | 1.5 (1.1, 2.0) | 1.6 (1.2, 2.1) |
| Initial | 2.2 (1.1, 4.3) | NA |
| Upslope (1 %/sec decrease in rate of reperfusion) | NA | 1.6 (1.1, 2.3) |
| Downslope (2 %/sec slowing in rate of decline) | 1.2 (1.0, 1.5) | NA |

^1^Stepwise Backward Elimination Technique Used. The p-value in parenthese after Yes is the p-value in the resultant model. The p-value after No is the p-value when the eliminated parameter was included back into the final model for re-evaluation after elimination.

^#^For patients with a history of chronic renal insufficiency or end stage renal disease, the total SOFA score used did not include the renal portion of the score.

**
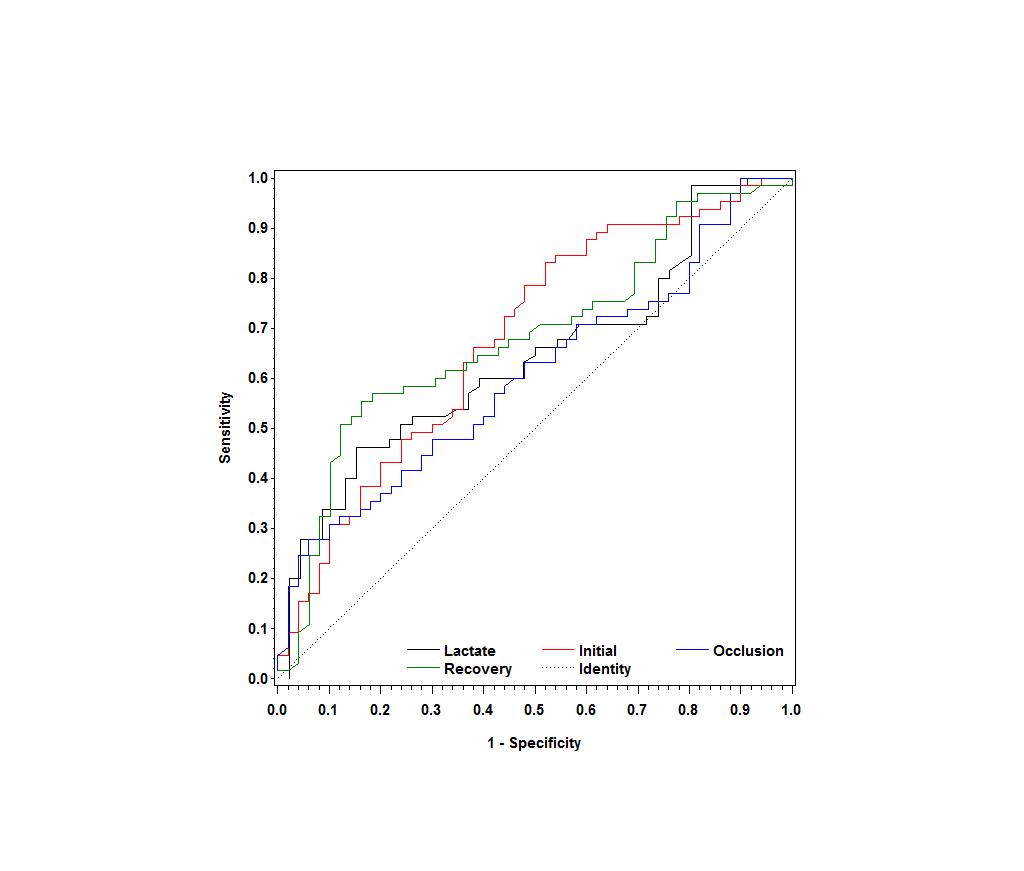
**

**A)**

**
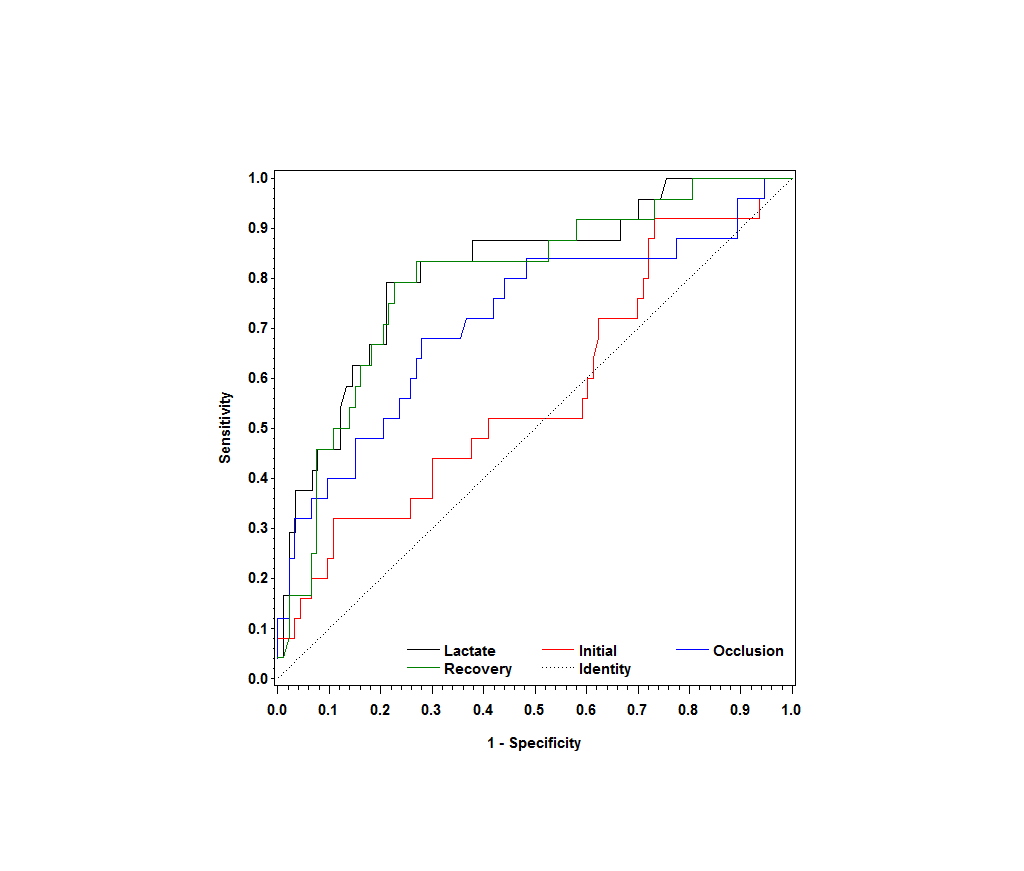
**

**B)**

**Figure 2A a) ROC for SOFA greater than 2 b) In-Hospital Mortality in Septic Shock and Sepsis Groups**
